# Impact of Long-Term Supplementation with Fish Oil in Individuals with Non-Alcoholic Fatty Liver Disease: A Double Blind Randomized Placebo Controlled Clinical Trial

**DOI:** 10.3390/nu12113372

**Published:** 2020-11-02

**Authors:** Kátia Cansanção, Marta Citelli, Nathalie Carvalho Leite, María-Carmen López de las Hazas, Alberto Dávalos, Maria das Graças Tavares do Carmo, Wilza Arantes Ferreira Peres

**Affiliations:** 1 Institute of Nutrition Josué de Castro of Federal University of Rio de Janeiro (UFRJ), Rio de Janeiro 21.941-902, Brazil; kcansancao@gmail.com (K.C.); tcarmo@editema.com.br (M.d.G.T.d.C.); 2 Instituto de Nutrição, Universidade do Estado do Rio de Janeiro, Rio de Janeiro 20559-900, Brazil; martacitelli@gmail.com; 3 Department of Internal Medicine, University Hospital Clementino Fraga Filho, School of Medicine of UFRJ, Rio de Janeiro 21.941-902, Brazil; nathaliecleite@gmail.com; 4 Laboratory of Epigenetics of Lipid Metabolism, Madrid Institute for Advanced Studies (IMDEA)-Food, CEI UAM+CSIC, 28049 Madrid, Spain; mcarmen.lopez@imdea.org (M-C.L.d.l.H.); alberto.davalos@imdea.org (A.D.)

**Keywords:** NAFLD, miRNA, miR-122, polyunsaturated fatty acid, fish oil, liver fibrosis, alkaline phosphatase

## Abstract

Non-alcoholic fatty liver disease (NAFLD) is a chronic disease affecting up to 25% of the population worldwide. n-3 long-chain polyunsaturated fatty acids (n-3 PUFA) have been associated with improved clinical parameters of NAFLD. Our purpose was to conduct a pilot study to evaluate the effects of n-3 PUFA supplementation in a randomized, double-blind, placebo-controlled clinical study performed on NAFLD individuals diagnosed by ultrasound. Patients received n-3 PUFA (*n* = 13) or placebo (*n* = 11) supplementation for six months. Circulating miR-122 expression (determined by quantitative real time-polymerase chain reaction (qRT-PCR), liver fibrosis (FibroScan^®^), red blood cells (RBC) fatty acids (gas chromatography), and biochemical tests were performed at baseline and after intervention. After the intervention, in the n-3 PUFA group, docosahexaenoic acid (DHA) and omega index increased significantly in RBC (*p* = 0.022 and *p* = 0.012, respectively), in addition to a significant reduction in alkaline phosphatase (ALP) (*p* = 0.002) and liver fibrosis (*p* = 0.039). However, there was no change in the expression of circulating miR-122 in both groups. Our results showed that omega-3 PUFA were incorporated in erythrocytes after six months of fish oil supplementary intake, and that n-3 PUFA were effective in reducing ALP and liver fibrosis without altering the expression of circulating miR-122 in individuals with NAFLD.

## 1. Introduction

Over the past few decades, non-alcoholic fatty liver disease (NAFLD) has emerged as the leading cause of chronic liver disease worldwide, affecting approximately 25% of the general population [1]. Its progression can lead to non-alcoholic steatohepatitis (NASH), and advanced liver fibrosis is considered an independent risk factor for mortality [2]. In addition, this complex multifactorial disease is related to several factors such as insulin resistance (IR), dysregulation of lipid metabolism, diet, inflammation, oxidative stress, intestinal microbiota, and genetic and epigenetic factors [3,4]. However, its current treatment is limited to controlling associated comorbidities and to healthier lifestyle changes, which are hard to maintain in the long-term [5,6].

In this sense, additional therapies have been investigated, and among the various compounds used [7,8], long-chain polyunsaturated fatty acids n-3 PUFA (eicosapentaenoic, EPA and docosahexaenoic, DHA) have shown promising results. In this sense, different formulations of EPA and DHA contribute to an improvement in metabolic, inflammatory, and histological profiles as well as in other relevant biochemical parameters [9,10,11,12,13,14,15,16]. In fact, even though the benefits are conferred to both fatty acids (FAs), DHA appears to be more effective in inhibiting the transcription of lipogenic enzymes via the suppression of the SREBP transcription factor, increasing FA oxidation via adenosine monophosphate-activated protein kinase (AMPK) activation and reducing pro-inflammatory cytokines by peroxisome proliferation-activated receptor alfa/gamma (PPARα/γ)-mediated suppression of CD36 [17]. Recently, n-3 PUFAs have been reported to modulate the expression of specific miRNAs [18,19,20].

These small non-coding RNAs have been extensively investigated as they regulate about 60% of all human genes. Among the several NAFLD-related miRNAs identified [21,22], miR-122 is the most abundant liver-expressed miRNA [23] and stands out as a potential non-invasive biomarker and therapeutic target of this liver disease [24]. miR-22 represents an important regulator of genes involved in lipid metabolism [25] and its deletion in vivo results in an indirect regulation of fatty acid synthase (FASN), acyl CoA carboxylase (ACC), (ACLY), stearoyl-CoA desaturase I (SCD1), and sterol regulatory element-binding protein 1 and 2 (SREBP1-2) genes as well as a reduction in plasma cholesterol and liver fat accumulation, and increased beta oxidation of fatty acids [25]. In addition, increased circulating miR-122 expression has been associated with the severity of NAFLD [26,27,28,29].

Currently, despite scientific advances, the plurality of factors related to NAFLD limit treatment strategies. Thus, considering the beneficial effects attributed to n-3 PUFA supplementation in previous studies, we hypothesized that the supplementation with these FA would modulate miR-122 expression in individuals with NAFLD, in addition to promoting an improvement in relevant biochemical and clinical parameters.

## 2. Materials and Methods 

### 2.1. Study Design and Participants

This was a randomized, double-blind, placebo-controlled clinical trial conducted between January 2018 and February 2020, which included patients attending the hepatology outpatient clinic of the Hospital Universitario Clementino Fraga Filho (HUCFF) (Rio de Janeiro, Brazil). Individuals of both sexes, diagnosed with ultrasound-confirmed NAFLD, aged 19 years old or older were included. However, those intaking more than 20 g of alcohol/day, using steroids, non-steroidal anti-inflammatory drugs, immunomodulatory agents, antibiotics, or n-3 PUFA supplementation in the 12 months prior to the study were excluded. In addition, individuals with viral hepatitis, human immunodeficiency virus (HIV), cancer, inflammatory bowel disease, chronic kidney disease, transplanted, pregnant and lactating women, with trauma, who had undergone surgery or had been hospitalized in the last 30 days were also excluded.

Randomization was performed using the system available at http://www.random.org, designed in 1:1 blocks by an independent pharmacist for the study. Everyone else including patients and researchers were blinded to the treatment provided. The secrecy of the randomization list was only accessed at the end of the intervention by all participants.

The protocol for this study was approved by the Research Ethics Committee of Hospital Universitario Clementino Fraga Filho (HUCFF), CAAE 68269317.9.0000.5257 and by the Brazilian Registry of Clinical Trials (ReBEC), number RBR-8dp876. The inclusion of each individual was made through formal authorization by signing a free and informed consent form, and in accordance with the guidelines of the Declaration of Helsinki.

Sample size was calculated based on the study by Cappani et al. [11], where biochemical improvements were seen in 64% of the patients supplemented with omega 3. Therefore, the following parameters were considered: significance level α = 5% (bilateral), power of the 1-β test = 80%, and expected difference in the absolute delta of the biochemical parameters between the relatively “large” groups (effect size > 1.0). According to GPower 3.1.9.2 for the Wilcoxon–Mann–Whitney test option, two independent groups were selected, with the minimum number of 18 cases per group. Therefore, the inability to reach a sufficient number of patients indicates that this study should be considered as a pilot study.

### 2.2. Interventions

n-3 PUFA capsules contained fish oil, vitamin E, gelatin, purified water, and glycerin as a humectant. Each capsule contained approximately 503 mg of DHA and 102 mg of EPA. As for the placebo, olive oil (Olea Europaea) was used, containing about 750 mg of oleic acid, in addition to smaller amounts of palmitic acid and linoleic acid (AL). The appearance of the capsules was similar. They were yellowish and soft, both weighing 1000 mg, without any coding, and maintaining double blinding. Each individual consumed three capsules (1.509 mg of DHA and 306 mg of EPA) per day for six months. 

### 2.3. Transient Hepatic Elastography

Steatosis and liver fibrosis were assessed by transient hepatic elastography using the FibroScan^®^ device (Echosens, Paris, France) at baseline and after six months of intervention. The examination was performed by an experienced and trained operator, with the patient having fasted for two hours. The procedure was performed with the patient in the supine position, with the right arm in maximum abduction, and the liver stiffness was measured between the sixth or seventh intercostal space in the right mid-axillary line. The exam was performed using the M or XL probe according to the feasibility of measuring liver stiffness [30], and only resulted in 10 valid measurements considered valid, with a success rate greater than 60% and interquartile range (IQR) of the value average stiffness less than or equal to 30%.

### 2.4. Sample Collection

Peripheral blood samples were obtained after 12-h overnight fasting using vacutainer tubes containing ethylenediamine tetraacetic acid (EDTA), which were centrifuged at 3000 rpm for 15 min to obtain plasma. Subsequently, the tubes containing erythrocytes were washed three times with sodium chloride (NaCl 0.9%, *w*/*v*), discarding the supernatant at the end of each centrifugation, and 100 μL of sodium dithionite (1.0% *w*/*v*) was added to the third wash. All biological fluids were stored in a −80 °C freezer until further analysis.

### 2.5. Biochemical Tests

Biochemical tests, alanine transaminase (ALT), aspartate aminotransferase (AST), ALP, GGT, fasting glucose, glycated hemoglobin (HbA1c), TG, CT, low-density lipoprotein (LDL), and high density lipoprotein (HDL) were obtained at baseline and after six months of treatment by consulting medical records. These tests are performed routinely as part of patient monitoring.

### 2.6. RNA Extraction, Real-Time PCR, and miR-122 Relative Expression

Total RNA was extracted from 200 μL of plasma using the miRNeasy serum/plasma kit (Quiagen, Hilden, Germany), following the manufacturer’s instructions. Subsequently, the TaqMan^®^ Advanced miRNA Assay Kit (Applied Biosystems, Foster City, CA, USA) was used for cDNA synthesis. Quantitative real-time was performed using the TaqMan^®^ Fast Master Mix [2x] and the specific primer for hsa-miR-122, in addition to the miR-451 used as a normalizer, in triplicate. The RT-qPCR reading was performed on a StepOnePlus System (Applied Biosystems, Thermo Fisher Brand, Foster City, CA, USA). The relative expression of miR-122 was calculated based on a calibrating control sample, after normalizing the values of the cycle threshold (CT) by the internal control miR-39. The results were analyzed using the ∆∆CT method, as previously described by [31].

### 2.7. Assessment of Fatty Acid Composition of Red Blood Cells (RBC)

RBC samples were subject to lipid extraction, saponification, and direct alkaline methylation using the adaptation of the method proposed by the American Oil Chemist’s Society AOCS 2b-11 (2017) [32]. The internal standard C13: 0 (Sigma-Aldrich, Saint Louis, MO, USA) and then 5 mL of sodium hydroxide (NaOH) in 0.5 M in methanol was added. The tubes were shaken and placed in a water bath for 15 min at 100 °C under agitation (4 rpm). After cooling the tubes, 5 mL of BF3-CH3OH (Sigma-Aldrich, Saint Louis, MO, USA) was added, mixed, and placed in a water bath at 100 °C with stirring for two minutes. After cooling the tubes, 5 mL of hexane and 3 mL of saturated NaCl were added. The samples were kept at RT for two hours for transesterification.

Following chromatographic conditions previously described [33], FAs were quantified in the GC 7890A chromatograph, equipped with a hydrogen flame ionization detector, and EZChrom Elite CDS software (Agilent Technologies, Inc., Santa Clara, CA, USA) using the capillary column SP 2560 (bisulopropyl polysiloxane, 100 m × 0.25 mm DI, thickness of 0.20 µm; Supelco, Bellefonte, PA, USA). The methyl esters of AGs were identified by comparing the retention time relative to the standards (Reference Standard GLC 463, Nu-Chek Prep, Inc., Elysian, MN, USA), which were expressed in the present study as a percentage. The omega 3 index was calculated by adding the % EPA and % DHA in RBC.

### 2.8. Anthropometric Assessment

The body mass index (BMI) was measured by dividing weight (kg) by height (m^2^). Waist circumference (WC) was measured between the midpoint of the last rib and the iliac crest using an inelastic measuring tape [34]. The body adiposity index (BAI) was calculated by the formula: [Hip/(height × √ height)] − 18 [35], and the waist–height ratio (WHR) was calculated by dividing the WC by the height in centimeters.

### 2.9. Statistical Analysis

The data were analyzed using the Statistical Package for the Social Sciences (SPSS) software, version 21.0. The hypothesis of normality was tested using the Shapiro–Wilk test. The Mann–Whitney test was used to compare the placebo group and n-3 PUFA. Categorical variables were analyzed using the chi-squared test. Within-group differences were assessed using the Wilcoxon signed-rank tests. A value of <0.05 was considered significant.

## 3. Results

### 3.1. Baseline Characteristics of the Subjects

**Figure 1:**
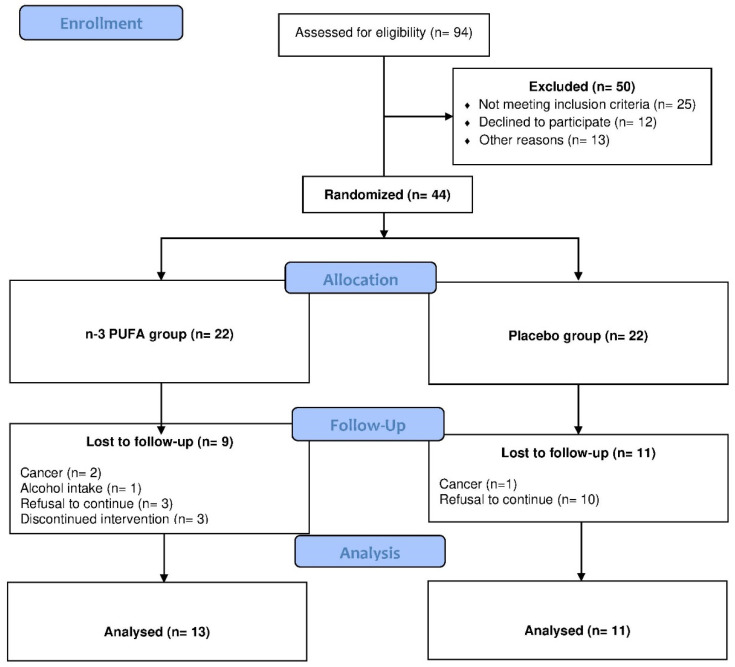
CONSORT flow chart of participant flow.

Twenty-four individuals completed the study out of the 44 selected for randomization. Of these, thirteen were allocated to the n-3 PUFA group and eleven to the placebo group. The main factors that led to segment loss were irregular capsule ingestion, refusal to continue, diagnosis of cancer during the study, diarrhea, and nausea (Figure 1).

The total population of this pilot study (*n* = 24) was composed predominantly of females (70.83%) with a median age of 57.5 years (51.00–67.00). Table 1 shows that, at baseline, the levels of demographic, anthropometric, and clinical variables showed no significant differences between the placebo and PUFA n-3 groups.

### 3.2. n-3 PUFA in RBC

**Figure 2:**
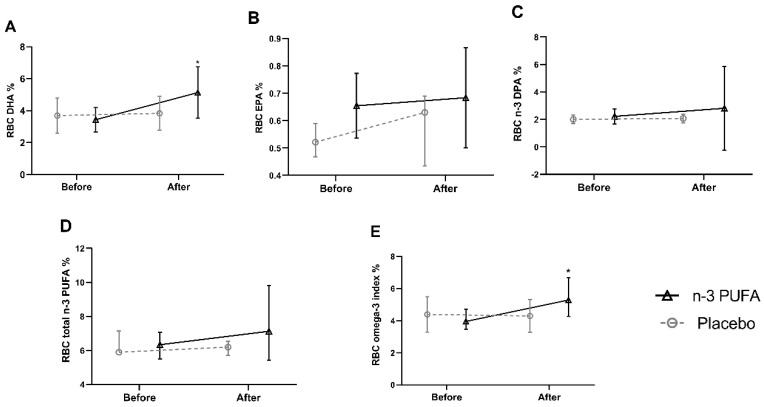
Percentage of DHA (**A**), EPA (**B**), n-3 DPA (**C**), total n-3 PUFAs (**D**), and omega-3 index (EPA + DHA) (**E**), in RBC, at baseline and after six months of intervention. Within-group differences were assessed using the Wilcoxon signed-rank test. Values are medians and interquartile ranges (IQRs). * *p* < 0.05 is considered statistically significant. DHA, docosahexaenoic acid; EPA, eicosapentaenoic acid; n-3 DPA, omega-3 docosapentaenoic acid; n-3 PUFA, omega-3 polyunsaturated fatty acid; RBC, red blood cell.

Compliance with supplementation was verified by comparing the percentage of red blood cell (RBC) FAs at baseline and after six months of intervention in both groups. Regarding RBC n-3 PUFAs, a significant increase was observed after six months of intervention in the percentage of DHA and omega-3 index in the treated group compared to the baseline [DHA; 3.314 (2.841–4.090) vs. 4.542 (3.765–5.991) (*p* = 0.022)], [omega-3 index; 3.949 (3.458–4.714) vs. 5.285 (4.275–6.670) (*p* = 0.012)], respectively. As for the EPA [0.611 (0.587–0.702) vs. 0.698 (0.556–0.824) *p* = 0.484], the n-3 docosapentaenoic acid (n-3 DPA) [2.152 (1.952–2.371) vs. 1.557 (1.237–2.041) *p* = 0.237], and total n-3 PUFA [(6.342 (5.492–7.067) vs. 7,127 (5.427–9.823) *p* = 0.345)], although statistical significance was not reached. In the placebo group, there was no significant change in the FAs (DHA, *p* = 0.767; EPA, *p* = 0.859; n-3 DPA, *p* = 0.401; total n-3 PUFA, *p* = 0.594; EPA + DHA *p* = 0.859) when comparing the baseline to six months of intervention (Figure 2). Additionally, capsule count was performed to confirm the adherence to supplementation.

### 3.3. Anthropometric, Biochemical, and Clinical Data

The comparison between anthropometric, biochemical, and clinical variables revealed significant reductions in serum ALP levels (*p* = 0.002) and in liver fibrosis, measured by transient hepatic elastography (THE) (*p* = 0.039), in the PUFA n-3 group after six months of intervention. In addition, a reduction in waist circumference (WC), gamma-glutamyl transferase (GGT), total cholesterol (CT), triglycerides (TG), and controlled attenuation parameter (CAP) were observed, although statistical significance was not reached. In the placebo group, there were no significant changes in the parameters evaluated (Table 2).

### 3.4. Evaluation of the Effects of Intervention on Circulating miR-122 Expression 

**Figure 3:**
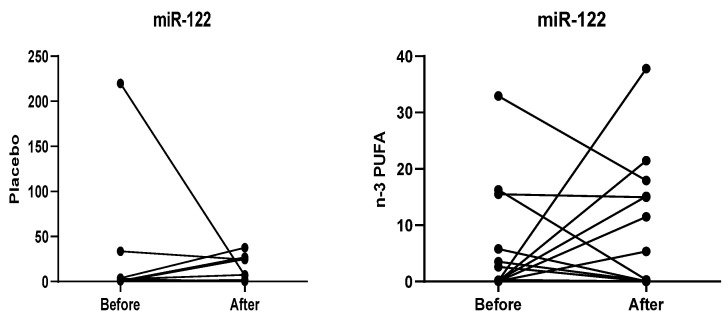
Comparison between the relative expression of miR-122 after six months of intervention. Within-group differences were assessed using the Wilcoxon signed-rank test.

The relative expression of miR-122 was not statistically significant (*p* > 0.05) when comparing baseline periods to six months of intervention, both in the placebo group (*p* = 0.721) and in the n-3 PUFA group (*p* = 0.807) (Figure 3). 

## 4. Discussion

This study showed that supplementation with fish oil including 1.509 mg of DHA and 306 mg of EPA to patients with NAFLD for six months influenced erythrocyte n-3 PUFA patterns and was accompanied by a decrease in ALP and LSM, measured by FibroScan^®^. These results are in accordance with other studies reporting that DHA was more effective in improving steatosis and liver fibrosis in children and adolescents [36,37]. 

The increase in DHA observed in the n-3 PUFA group suggests adherence to the supplementation protocol was properly complied. In addition, as there was no difference between n-3 PUFA in the placebo group, we considered that the influence of other dietary factors, which could potentially skew the results, was not significant. Corroborating these results, the omega-3 index, used as a marker of n-3 PUFA intake in the medium and long term [38], increased only in the treated group, after six months. Similar results were found previously by Tobin et al. [15], who demonstrated a significant increase in the omega index in the intervention group compared to the placebo group after 24 weeks of follow-up.

Unlike other studies reporting a reduction in the hepatic enzymes ALT, AST, or GGT [13,14,15,16,17,18,19,20], after the supplementation of n-3 PUFA, we found a reduction in the levels of ALP, which is commonly used as a marker of hepatobiliary and bone diseases [39,40], and has recently been described as an independent predictor of NAFLD in older women [41]. In addition, significantly higher levels of ALP have been demonstrated in NAFLD patients in stages 1 and 2 of liver fibrosis compared to individuals without fibrosis [42]. However, even though liver and bone tissues are the main contributors to the circulating pool, it should be noted that different tissues secrete this enzyme, and that other ALP isoforms should be evaluated [43].

Here, a decrease in liver fibrosis was measured in the n-3 PUFA-supplemented group after six months of intervention. Instead of a liver biopsy, the gold standard for assessing liver fibrosis, FibroScan^®^, was the method of choice here and our findings were similar to the previous results described by Li et al., which demonstrated a significant reduction in liver fibrosis, assessed by histology, after six months of intervention with n-3 PUFA [16]. It is noteworthy that recent studies have shown that liver stiffness measured by FibroScan^®^ has good accuracy compared to liver biopsy, regardless of the type of probe used, the degree of liver steatosis, and inflammation [30,44,45].

The decrease in liver fibrosis observed in the treated group might be due to the modulation of DHA-mediated inflammation. In this sense, in previous publications, supplementation of n-3 PUFA to NAFLD patients was associated with a reduction in cytokines and pro-inflammatory eicosanoids [12,13,14]. In an animal model, this lipid class suppressed the expression of caspase 3, which is related to endoplasmic reticulum stress and apoptosis [46]. However, as no inflammatory parameter was evaluated here, this hypothesis could not be confirmed. 

As far as we know, this was the first double-blinded, placebo-controlled clinical study evaluating the effects of n-3 PUFA on miR-122 expression. Our findings did not demonstrate modulation of this circulating miRNA after six months of intervention. In contrast, Baselga-Escudero et al. observed a positive regulation of miR-122 in the mononuclear cells of obese rats with dyslipidemia, induced by a cafeteria and standard diet. Furthermore, after treatment with DHA, there was a negative regulation of this miRNA in liver tissue as well as FAS mRNA, and increased peroxisome proliferator-activated receptor (PPARβ/γ) [47]. These discrepancies could be justified by two factors.

First, the difference in biological material used for comparison in the previous study could skew these results, since miRNA expression varies between tissues [48]. Corroborating this statement, circulating levels of miR-122 have been shown to be high compared to healthy controls, whereas in liver tissue, the opposite is observed [49,50,51]. Second, there is a high variability in the expression of circulating miR-122, both interindividual and intraindividually, as recently demonstrated by Vogt et al. and Church and collaborators [52,53]. These two recent studies have associated the high variation of miR-122 with ethnicity. Here, Brazilian individuals with a great racial miscegenation were comprised, which could help explain the variability observed in the expression of this miR, resulting in its non-significant modulation.

### Strengths and Limitations

Our study design had limitations and strengths. The major limitation is the reduced number of patients due to the large dropout from the clinical trial. Nevertheless, this is the first Brazilian study on NAFLD patients supplemented with DHA-rich fish oil that aimed at evaluating the clinical and biomolecular effects produced and the incorporation of these fatty acids. Therefore, this study provides an overview on the n-3 PUFAs status of Brazilian NAFLD patients and may contribute, in the future, to the establishment of effective dosages and duration of EPA and DHA supplementation methods.

## 5. Conclusions

In conclusion, our pilot study provides evidence that omega-3 PUFA, in particular DHA, are incorporated in erythrocytes after six months of fish oil supplementation in patients with NAFLD. We also showed that DHA supplementation was effective in reducing ALP and liver fibrosis, suggesting that the dosage and duration of omega-3 PUFA supplementation used here were capable of ameliorating the liver damage occurring in NAFLD patients. We believe that future larger trials to confirm these encouraging results are warranted. Even though the expression of circulating miR-122 was not affected under these conditions, further screening for additional small non-coding RNAs with potential regulatory roles in pathological milieus should be conducted as the search for new therapeutic targets is incessant. 

## Figures and Tables

**Table 1:** Demographic, clinical, and biochemical variables of patients with non-alcoholic fatty liver disease (NAFLD) included in the study.

Variables	Placebo Group (*n* = 11)		n-3 PUFA Group (*n* = 13)		*p* Value
Sex, M/F	8/3		9/4		0.851
Age, yr	55.00	52.00–74.00	62.00	52.00–68.00	0.910
BMI (kg/m^2^)	31.20	28.82–35.19	29.62	26.60–35.79	0.691
WC (cm)	104.90	98.00–112.50	109.00	91.50–116.00	0.955
BAI	34.62	30.60–40.12	39.31	32.05–42.20	0.277
WHtR	0.63	0.57–0.65	0.68	0.55–0.72	0.820
ALT (U/L)	30.00	22.00–32.00	24.00	16.00–41.00	0.494
AST (U/L)	20.00	18.00–24.00	27.00	16.00–33.00	0.531
GGT (U/L)	42.00	26.00–62.00	53.00	35.00–88.00	0.150
ALP (U/L)	78.00	52.00–114.00	92.00	58.00–140	0.392
Fasting glucose (mg/dL)	147.00	96.00–186.00	100.00	96.00–146.00	0.167
HbA1c %	7.30	5.90–8.20	7.20	5.80–7.70	0.608
TC (mg/dL)	187.00	146.00–203.00	197.00	155.00–247.00	0.252
LDL (mg/dL)	91.00	62.00–125.00	87.00	72.00–160.00	0.531
HDL (mg/dL)	46.00	40.00–49.00	47.00	39.00–59.00	0.608
TG (mg/dL)	166.00	104.00–260.00	182.00	121.00–241.00	1.000
CAP baseline (dB/m)	271.00	239.00–334.00	330.00	281.00–369.00	0.167
Liver fibrosis (kPa)	5.00	4.00–5.60	6.00	5.00–7.00	0.072

Data are medians and IQR. The Mann–Whitney test was used for numerical variables and the chi-square (X2) test was used for categorical variables. Difference between the placebo and omega 3 groups: p < 0.05. IQR, interquartile range; BMI, body mass index; WC, waist circumference; WHR, waist/hips ratio; BAI, body adiposity index; WHtR, waist/height ratio; ABSI, body shape index; ALT, alanine transaminase; AST, aspartate transaminase; GGT, gamma-glutamyl transferase; ALP, alkaline phosphatase; HbA1c, Glycated hemoglobin; LDL, low-density lipoprotein; HDL, high-density lipoprotein; TC, total cholesterol; TG, triglycerides; CAP, controlled attenuation parameter; kPa, kilopascals.

**Table 2:** Changes in anthropometric, biochemical, and clinical outcomes of non-alcoholic fatty liver disease patients after the intake of n-3 PUFA or placebo for six months.

	Placebo Group (*n* = 11)					n-3 PUFA Group (*n* = 13)				
Variables	Baseline		Post-Treatment		*p* Value	Baseline		Post-Treatment		*p* Value
	Median	IQR	Median	IQR		Median	IQR	Median	IQR	
BMI (kg/m^2^)	31.20	28.82–31.19	31.39	27.76–35.17	0.799	32.89	28.26–37.18	32.84	28.27–37.17	0.917
WC (cm)	104.90	98.00–112.50	105.00	97.50–113.00	0.476	111.00	93.00–116.25	99.50	92.50–120.00	0.906
BAI	34.62	30.60–40.12	35.11	31.41–39.44	0.130	39.85	34.02–44.31	38.86	33.48–44.93	0.432
WHtR	0.63	0.57–0.65	0.64	0.56–0.66	0.633	0.69	0.56–0.72	0.66	0.55–0.73	0.751
ALT (U/L)	30.00	22.00–32.00	31.00	22.00–39.00	0.540	30.00	18.50–42.50	35.50	17.00–42.00	0.753
AST (U/L)	20.00	18.00–24.00	24.00	17.00–25.00	0.644	30.00	17.00–33.50	29.00	21.00–32.50	0.889
GGT (U/L)	42.00	26.00–62.00	34.00	22.00–45.00	0.139	50.00	35.50–70.00	42.50	30.00–57.00	0.382
ALP (U/L)	78.00	52.00–114.00	61.00	47.00–79.00	0.333	93.00	70.50–157.50	57.00	44.75–73.50	0.002 *
Fasting glucose (mg/dL)	147.00	96.00–186.00	108.00	100.00–147.00	0.102	100.00	96.00–143.50	119.50	89.50–157.00	0.132
HbA1c %	7.30	5.90–8.20	6.90	6.20–7.70	0.332	6.30	5.80–7.75	6.60	5.90–8.84	0.694
TC (mg/dL)	187.00	146.00–203.00	155.00	144.00–182.00	0.445	197.00	152.50–247.50	167.50	131.25–180.00	0.115
LDL (mg/dL)	91.00	62.00–125.00	79.00	67.00–95.00	0.445	87.00	74.50–160.50	84.50	63.25–92.50	0.162
HDL (mg/dL)	46.00	40.00–49.00	46.00	36.00–51.00	0.474	47.00	41.50–59.50	45.50	42.00-–52.00	0.238
TG (mg/dL)	166.00	104.00–260.00	126.00	119.00–224.00	0.203	168.00	118.00–226.50	147.50	91.75–192.50	0.208
CAP (dBm/min)	271.00	239.00–334.00	310.00	267.00–354.00	0.374	330.00	281.00–369.50	313.00	251.00–337.00	0.060
Liver fibrosis (kPa)	5.00	4.00–5.60	5.60	4.40–6.20	0.211	6.80	5.60–7.60	6.10	4.55–7.40	0.039 *

Values are medians and interquartile ranges (IQRs). * Difference between baseline and after six months of intervention in the placebo and PUFA n-3 groups; *p* < 0.05 was considered significant. The Wilcoxon test was applied to numerical variables. BMI, body mass index; WC, waist circumference; WHR, waist/hips ratio; BAI, body adiposity index; WHtR, waist/height ratio; ABSI, body shape index; ALT, alanine transaminase; AST, aspartate transaminase; GGT, gamma-glutamyl transferase; ALP, alkaline phosphatase; HbA1c, Glycated hemoglobin; TC, total cholesterol; TG, triglycerides; LDL, low-density lipoprotein; HDL, high-density lipoprotein; CAP, controlled attenuation parameter; kPa, kilopascals.
